# Neuroprotective Effect of Catalpol *via* Anti-Oxidative, Anti-Inflammatory, and Anti-Apoptotic Mechanisms

**DOI:** 10.3389/fphar.2020.00690

**Published:** 2020-05-14

**Authors:** Chunjing Yang, Zhengyuan Shi, Longtai You, Yuanyuan Du, Jian Ni, Dan Yan

**Affiliations:** ^1^Department of Pharmacy, Beijing Shijitan Hospital, Capital Medical University, Beijing, China; ^2^Beijing Key Laboratory of Bio-characteristic Profiling for Evaluation of Rational Drug Use, Beijing, China; ^3^International Cooperation & Joint Laboratory of Bio-characteristic Profiling for Evaluation of Rational Drug Use, Beijing, China; ^4^School of Chinese Materia Medica, Beijing University of Chinese Medicine, Beijing, China; ^5^Beijing Research Institute of Chinese Medicine, Beijing University of Chinese Medicine, Beijing, China

**Keywords:** anti-inflammation, anti-oxidative, catalpol, nuclear factor-κB, Kelch-like ECH-associated protein 1/Nuclear factor E2-related factor 2

## Abstract

Neuroinflammation and neuro-oxidative damage are now considered to be key factors in the neurological diseases. Therefore, it is important to study anti-inflammatory and neuroprotective agents. The present study investigated the anti-inflammatory and neuroprotective effects of catalpol (CAT), and the potential molecular mechanisms involved. The findings revealed that CAT markedly downregulated pro-inflammatory mediator nitric oxide (NO) and cytokines, including interleukin (IL)-6 and tumor necrosis factor (TNF)-a in lipopolysaccharide (LPS)-treated BV2 microglial cells. Moreover, CAT significantly decreased the levels of intracellular reactive oxygen species (ROS) and malondialdehyde (MDA), increased superoxide dismutase (SOD) activity and glutathione (GSH) level, reversed apoptosis, and restored mitochondrial membrane potential (MMP) in primary cortical neurons stimulated with hydrogen peroxide (H_2_O_2_). Furthermore, mechanistic studies showed that CAT inhibited nuclear factor-κB (NF-κB) pathway and p53-mediated Bcl-2/Bax/casaspe-3 apoptotic pathway. Moreover, it targeted the Kelch-like ECH-associated protein 1(Keap1)/Nuclear factor E2-related factor 2 (Nrf2) pathway. In summary, CAT may exert neuroprotective potential by attenuating microglial-mediated neuroinflammatory response through inhibition of the NF-κB signaling pathway. It blocked cortical neuronal oxidative damage by inhibiting p53-mediated Bcl-2/Bax/casaspe-3 apoptosis pathway and regulating Keap1/Nrf2 pathway. These results collectively indicate the potential of CAT as a highly effective therapeutic agent for neuroinflammatory and neuro-oxidative disorders.

## Introduction

Inhibition of neuroinflammation and neuro-oxidation is a critical action mode in the treatment of neurological diseases such as Alzheimer's disease, Parkinson's disease, and Huntington's disease (Kim et al., 2018; Mesika and Reichmann, 2019). Microglia act as important innate immune cells in the central nervous system (Kim et al., 2017). At rest, microglia are scavengers in the central nervous system to remove damaged neurons and pathogens. However, upon stress and injury, microglia produce neurotoxic molecules, which induce neuronal cell damage and the production of neurodegenerative diseases. Neuron cell membranes have high levels of unsaturated fatty acids, high oxygen consumption, and weak antioxidant defense capacity. Therefore, oxidative stress is extremely susceptible to damage to the nervous system, resulting in neurodegenerative diseases (Islam, 2017; Singh et al., 2019). The cerebral cortex is a superior center that regulates or controls body movement, and is involved in functional roles in cognition, learning, and memory (Frey et al., 1993). Primary cortical neurons are cells extracted from the cerebral cortex. They are one of the major cells in the cerebral cortex. In recent years, a growing number of studies have shown that damage to cortical neurons caused by oxidative stress is involved in the development of many neurological diseases, such as Alzheimer's disease and Parkinson's disease (Dinda et al., 2019; Hajizadeh Moghaddam et al., 2020). Therefore, it has become a novel strategy to find a potential drug with multi-target and multi-pathway neuroprotective effect in the treatment of neurological diseases.

**Figure 1:**
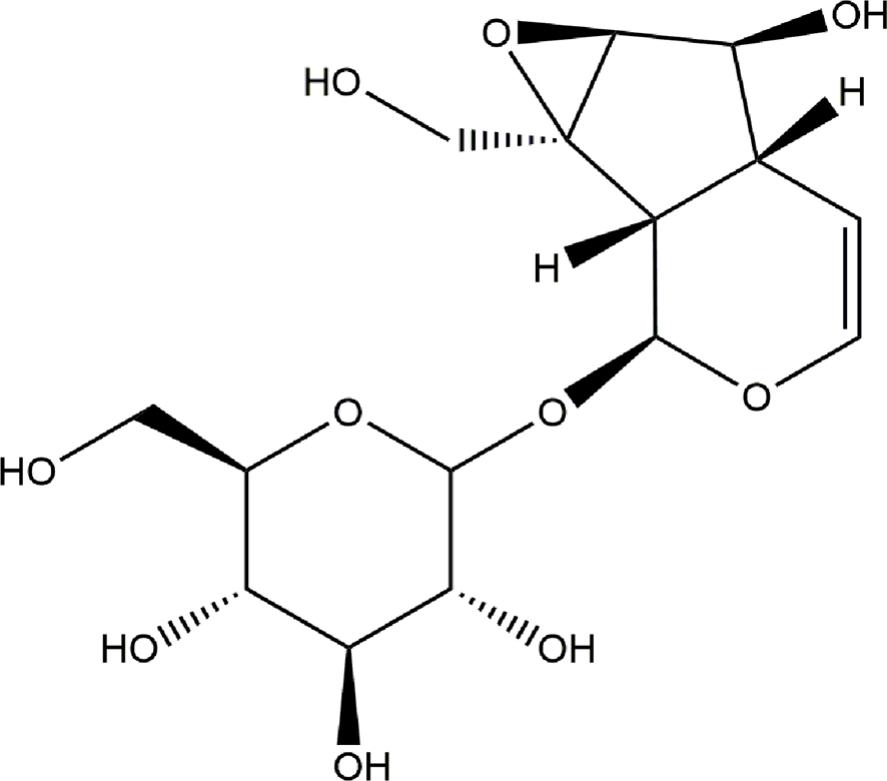
Chemical structure of CAT.

Catalpol (CAT), an iridoid glycoside compound, is isolated from Radix *Rehmanniae*. Numerous studies have suggested that CAT exhibits anti-inflammatory and anti-oxidative effects. *In vitro*, it attenuates hydrogen peroxide (H_2_O_2_)-induced apoptosis of PC12 cells (Jiang et al., 2004). Moreover, the antioxidant property of CAT has been well studied in gerbils (Li et al., 2004). It has been reported that CAT inhibited the production of free radicals and promoted antioxidant capacity in human umbilical vein endothelial cells (Hu et al., 2010). Studies have also shown that CAT enhanced recovery of cerebral function after cerebral ischemia, through a mechanism involving anti-inflammation (Zhu et al., 2015). However, the anti-neuroinflammatory and anti-oxidative effects of CAT on BV2 microglia and primary cortical neuron cells remain unclear. Therefore, the aim of the present study was to investigate whether CAT could protect BV2 microglia from lipopolysaccharide (LPS)-induced inflammation, and whether it could protect primary cortical neuron cells from H_2_O_2_-mediated oxidative stress. The oxidative parameters determined in appropriate cell models were reactive oxygen species (ROS), mitochondrial membrane potential (MMP), glutathione (GSH), malondialdehyde (MDA), and superoxide dismutase (SOD), while the inflammatory indices were nitric oxide (NO), tumor necrosis factor (TNF)-α, and interleukin (IL)-6. The chemical structure of CAT is shown in **Figure 1**.

## Materials and Methods

### Drugs and Chemicals

Catalpol (CAT; ≧98% purity) was provided by Shanghai Yuanye Bio-Technology Co. Ltd. (Shanghai, China). Dulbecco's modified eagle's medium/F-12 (DMEM/F-12) and fetal bovine serum (FBS) were bought from Corning (NY, USA). Hanks' balanced salt solution (HBSS), minimum essential medium (MEM), glucose solution, neurobasal medium, B27, L-glutamine, and trypsin were purchased from Life Technologies (NY, USA). Horse serum was bought from Hyclone (Logan, Utah, USA). LPS (from *E. coli*, isotype 055:B5) was obtained from Sigma Chemical Co. (St. Louis, MO, USA), while H_2_O_2_ was provided by Beijing Chemical Works (Beijing, China). Phosphate buffered saline (PBS), dimethyl sulfoxide (DMSO), and 3-(4, 5 -dimethylthiazol-2-yl)-2, 5-dipheny-ltetrazolium bromide (MTT) were obtained from Solarbio (Beijing, China). NO assay kit was provided by Applygen Co. (Beijing, China). TNF-α and enzyme-linked immunosorbent assay (ELISA) kits for IL-6 were purchased from BOSTER (Wuhan, China). Annexin V-FITC apoptosis, DAPI, ROS, and MMP were supplied by Beyotime (Nanjing, China). MDA, SOD, and GSH were obtained from Jiancheng (Nanjing, China). The antibodies for Bax, Bcl-2, caspase 3, cleaved-caspase 3, p53, Kelch-like ECH-associated protein 1 (Keap1), Nuclear factor E2-related factor 2 (Nrf2), quinone oxidoreductase 1 (NQO1), heme oxygenase-1 (HO-1), and NF-κB p65 and IκB-α and the phosphorylated forms of nuclear factor-κB (NF-κB) p65, IκB-α were products of Cell Signaling Technology (Massachusetts, USA).

### BV2 Microglia Cell Culture

The BV2 microglial cells (Chinese Academy of Sciences, Shanghai, China) were cultured in high glucose DMEM/F-12 medium containing 10% heat-inactivated FBS and 1% penicillin (10,000 U/ml) and streptomycin (10,000 μg/ml) in a humidified atmosphere with 5% CO_2_ at 37°C

### Isolation and Culture of Primary Cortical Neurons

All animal protocols used in this study were approved by the animal Care and Use Committee of the Beijing Shijitan Hospital. Primary cortical neuron cells were obtained from the brain of E14 C57BL/6 mouse embryos bought from SPF Biotechnology Co. Ltd., Beijing, China. Dissected cortical tissue from embryonic 14-day old mouse was minced and incubated with 0.125% trypsin in Ca^2+^- and Mg^2+^-free HBSS for about 20 min at 37°C. Then, the trypsin was carefully removed and the plating media (MEM media supplemented with 20% horse serum, 0.5 mM glutamine, 1.35 g glucose, 1% penicillin-streptomycin) was added to re-suspend the cells. Tissue chunks were removed using a 40 μM cell strainer. The single-cell suspension was cultured on poly-D-lysine-coated plates in the plating media. After 4–8 h, the plating media was replaced with the feeding medium i.e. neurobasal medium supplemented with 2% B27, 0.5 mM glutamine, 1% penicillin (10,000 U/ml), and streptomycin (10,000 μg/ml). Half of the medium was aspirated from each well and replaced with fresh feeding medium (warmed to 37°C) every 2 days until the neurons were mature (Beaudoin et al., 2012).

### Cell Viability Assay

The effect of CAT on the viabilities of BV2 microglia and primary cortical neurons was determined using MTT assay. The BV2 microglial cells were plated into 96-well plates at a density of 2.0 × 10^5^ cells/ml. After 24 h, the cells were pre-treated for 24 h with CAT at concentrations of 0, 1, 5, 25, 50, and 100 μM. The control was treated with DMSO (0.05%) in place of CAT. After 24 h, 100 μl of MTT working solution (0.5 mg/ml) was added to each well. After 2–4 h, the culture supernatant was replaced with 100 μl of DMSO to dissolve the purple formazan crystals formed. The optical density (OD) of each formazan solution was read at 570 nm in a microplate reader (Thermo, Multiskan GO, USA).

Primary cortical neurons were cultured in 96-well plates at a density of 1.0 × 10^4^ cells/well. On the seventh day, the cells were treated with CAT at concentrations of 0, 12.5, 25, 50, and 100 μM, and also with different concentrations of H_2_O_2_ for 24 h. Cells in the untreated control received DMSO (0.1%). After 24 h, 100 μl of MTT working solution (0.5 mg/ml) was added to each well. Following incubation for 2–4 h, the culture supernatant was replaced with 100 μl of DMSO to dissolve the resultant purple formazan crystals. The OD of each formazan solution was read at 570 nm in a microplate reader (Thermo, Multiskan GO, USA).

### Assay of NO Production

NO production was measured using NO assay kit based on the Griess reaction. The BV2 microglial cells (2.0 × 10^5^ cells/ml) were cultured in 96-well plates and pretreated with CAT for 2 h before treatment with LPS (0.5 μg/ml). After 18 h, NO level was measured in the culture supernatant using the Griess reaction. The OD of the mixture was read at 540 nm in a microplate reader.

### ELISA Assays

The effect of CAT on the expressions of TNF-α and IL-6 in the culture supernatant was determined with ELISA kits. In this assay, BV2 microglia were cultured in 96-well plates at a density of 1.0 × 10^5^ cells/ml, and were pretreated with CAT for 2 h before treatment with LPS (0.5 μg/ml). After incubation for 18 h, the expression of TNF-α and IL-6 in the culture supernatant were determined using their corresponding ELISA kits in line with the manufacturers' instructions. The OD of the mixture was read at 450 nm in a microplate reader.

### Measurement of Intracellular ROS

ROS are produced at the beginning of the inflammation phase. Therefore, the effect of CAT on intracellular ROS accumulation was measured. The generation of intracellular ROS was determined using DCFH-DA fluorescent dye (Wang and Joseph, 1999). The DCFH-DA probe is a non-polar compound which lightly diffuses into cells, where it is hydrolyzed by intracellular esterase to generate DCFH which is trapped within the cells and becomes intracellularly oxidized to form the highly fluorescent compound 2, 7-dichlorofluorescein (DCF) which is measured flow cytometrically. In this assay, primary cultured cortical neurons were seeded in six-well plates. Following various treatments, the cells were incubated with 10 μM DCFH-DA for 30 min at 37°C in the dark. Subsequently, the cells were harvested, washed twice with PBS, and re-suspended for fluorescence analysis using a flow cytometer.

### DAPI Staining

Apoptosis was measured with DAPI staining (Lin et al., 2015). The primary cultured cortical neurons were fixed with 4% paraformaldehyde for 10 min after treatments. Then, the cells were washed with PBS and permeabilized using DAPI staining solution for 5 min. Thereafter, the cells were washed with PBS and examined under fluorescence microscope.

### Annexin V-FITC and PI Double Staining

The apoptotic cells in each group were treated with CAT at doses of 12.5, 25, and 50 μM before stimulation with H_2_O_2_ (50 μM) for 2 h. Then, the apoptotic cells were determined using Annexin V-Alexa FITC detection kit, according to the manufacturer's protocol. Fluorescence was measured with flow cytometry (You et al., 2018).

### Assay of MMP

Changes in MMP were determined with JC-1 by measuring red *vs.* green ﬂuorescence (healthy cells with high MMP give out red ﬂuorescence [JC-1-aggregate], while apoptotic/dead cells emit green ﬂuorescence [JC-1-monomer] because of lack of MMP). Primary cultured cortical neurons were cultured in six-well plates and treated with different concentrations of CAT (50, 25, and 12.5 μM) before stimulation with H_2_O_2_ (50 μM) for 2 h. The cells were then harvested *via* trypsinization, and washed with PBS. Thereafter, the cells were exposed to JC-1 staining solution, incubated for 20 min at 37°C in a CO_2_ incubator, washed with PBS to remove unbound dye, and re-suspended in PBS. The fluorescence intensity was measured with flow cytometry.

### Measurement of Levels of GSH, MDA, and SOD

The generation of GSH was determined with GSH assay kit (Jiancheng, Nanjing, China). Primary cultured cortical neurons were cultured in six-well plates and treated with different concentrations of CAT (50, 25, and 12.5 μM) before stimulation with H_2_O_2_ (50 μM) for 2 h. The cells were then harvested *via* trypsinization, washed with PBS, and broken up using ultrasound cell breaker. The resultant cell suspension was used for measurement of GSH, MDA, and SOD with assay kits, in accordance with the manufacturer's instruction.

### Western-Blot

The cells were lysed using RIPA (Beijing Dingguo Changsheng Biotechnology Co. Ltd.). The concentration of total protein in the lysate was determined with bicinchoninic acid (BCA) assay. Protein samples were subjected to 10% SDS–polyacrylamide gel electrophoresis. The proteins were electro-transferred from the gels to PVDF membranes to form blots.

The membranes were blocked with 5% (v/v) dried milk and incubated at 4˚C overnight with anti-Bax, anti-Bcl-2, anti-caspase 3, anti-cleaved caspase 3, anti-p53, anti-Keap1, anti-Nrf2, anti-NQO1, anti-HO-1 (Cell Signaling Technology, USA), anti-p-NF-κB p65, anti- NF-κB p65, anti-IκBα, and anti-p-IκBα (Cell Signaling Technology, USA). Thereafter, the membranes were incubated with HRP-conjugated goat anti-mouse IgG for 1 h at room temperature. β-Actin and GAPDH (Cell Signa ling Technology, Inc.) were used as the reference proteins.

### Statistical Analysis

Each experiment was repeated at least in triplicate and data were shown as mean ± SD. The data obtained were subjected to statistical analyses using SPSS 17.0 software. Statistical differences were determined with one-way ANOVA and LSD test. Differences were considered significant at ^*^p < 0.05, ^**^p < 0.01 *vs.* H_2_O_2_; *^#^p* < 0.05, *^##^p* < 0.01 *vs.* control.

## Results

### Effect of CAT and H_2_O_2_ on Cell Viability

**Figure 2:**
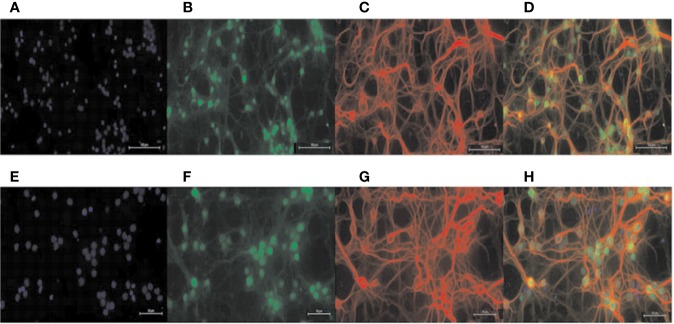
Identification of primary neurons. DAPI staining labeled all nuclei **(A, E)**; NEUN staining labeled primary neuron nuclei **(B, F)**; MAP-2 staining labeled primary neuron dendrites **(C, G)**; merged Figure **(D, H)**. Scale = 100 μM **(A–D)**; scale = 50 μM **(E–H)**.

**Figure 3:**
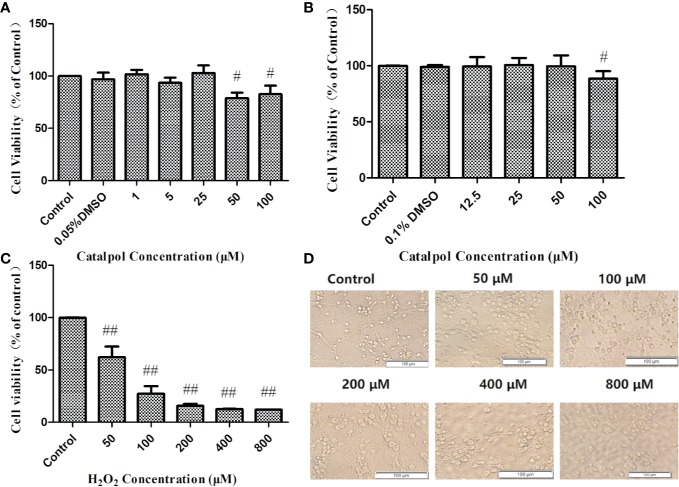
Cell viability of BV2 **(A)** and primary neurons **(B)** treated with CAT in three independent experiments. Cell viability of primary cortical neurons treated with H_2_O_2_ and the corresponding morphology **(C, D)**. MTT assay data are presented as mean ± SD. (^#^*p* < 0.05, ^##^*p* < 0.01 *vs.* Control).

Normal primary cortical neurons (**Figure 2**) were used for the next experiments. In order to eliminate experimental errors caused by non-specific cytotoxicity, cell viability testing was carried out. Compared with the control group, CAT at concentrations lower than, or equal to 25 μM had no effect on the viability of BV2 microglia. Thus, CAT was used in subsequent experiments at concentrations of 1, 5, and 25 µM (**Figure 3A**). Moreover, CAT at concentrations lower than, or equal to 50 μM had no effect on the viability of primary cortical neurons. Thus, CAT was used in subsequent studies at concentrations of 12.5, 25, and 50 µM (**Figure 3B**).

The neuronal viabilities were 62 and 27% at H_2_O_2_ concentrations of 50 and 100 µM, respectively. When the cells were stimulated with 50 µM H_2_O_2_ for 2 h, they became swollen and rounded, and there were many cell fragments. Moreover, the interwoven cellular network disappeared. The higher the concentrations of H_2_O_2_, the more the degree of cell death. Therefore, 2 h of stimulation with H_2_O_2_ (50 µM) was used in subsequent studies (**Figures 3C, D**).

### CAT Inhibited LPS-Induced NO, TNFα, and IL-6 Production in BV2 Microglia

**Figure 4:**
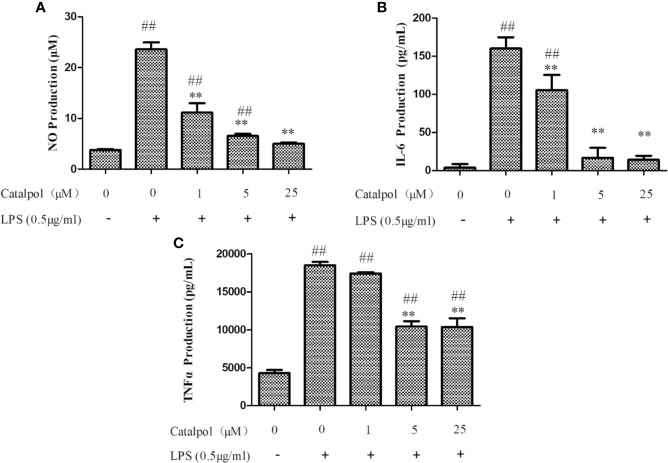
Effect of CAT on levels of NO **(A)**, IL-6 **(B)**, and TNF-α **(C)** in LPS-stimulated BV2 microglial cells. BV2 microglia were pretreated with different concentrations of CAT (1, 5, and 25 μM) for 2 h before treatment with LPS (0.5 μg/ml) for 18 h. Data are presented as mean ± SD of three independent experiments (^**^p < 0.01 *vs.* LPS; ^##^*p* < 0.01 *vs.* control).

In order to study the effect of CAT on LPS-induced inflammatory responses, and the molecular mechanisms involved, the ability of CAT to regulate NO production in response to LPS stimulation was first investigated. The LPS-stimulated group exhibited significantly increased NO production, when compared with the control group. However, as shown in **Figure 4A**, CAT significantly suppressed NO production (11.13, 6.55, and 5.02 μM) at 1, 5, and 25 μM, respectively. Thus, CAT treatment inhibited LPS-induced NO production in a dose-dependent manner (1–25 μM).

Based on inhibitory effect of CAT on NO production, the regulatory effect of CAT on pro-inflammatory cytokines (TNF-α and IL-6) in BV2 microglia was determined. Stimulation with LPS markedly induced the expression of IL-6 in BV2 microglia. In contrast, CAT significantly downregulated IL-6 expression (105.44, 16.88, and 14.17 pg/ml at CAT doses of 1, 5, and 25 μM, respectively; **Figure 4B**). As shown in **Figure 4C**, LPS stimulation markedly increased the expression of TNF-α in BV2 microglia. However, CAT significantly inhibited TNF-α production (10441 and 10359 pg/ml at doses of 5 and 25 μM, respectively). Thus, CAT treatment markedly inhibited LPS-induced expressions of IL-6 and TNF-α, indicating its good ability of anti-inflammatory effect.

### CAT Reversed H_2_O_2_-Induced ROS and MDA Production, and GSH and SOD Reduction

**Figure 5:**
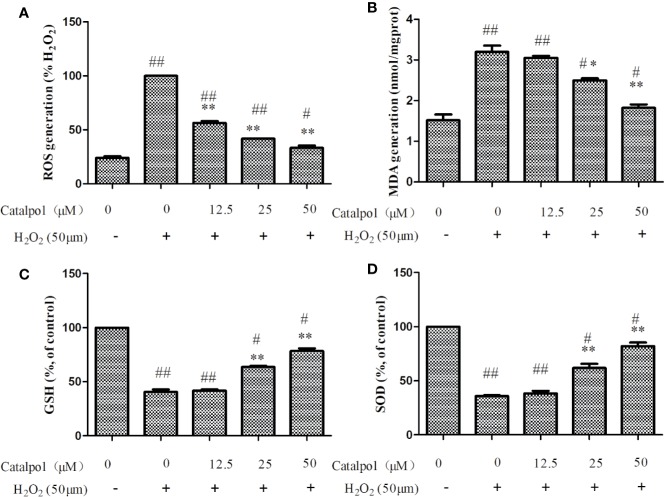
Effect of CAT on levels of ROS **(A)**, MDA **(B)**, GSH **(C)**, and SOD **(D)** in H_2_O_2_-stimulated primary cortical neurons. Primary cortical neurons were pretreated with different concentrations of CAT (12.5, 25, and 50 μM) before stimulation with H_2_O_2_ (50 μM) for 2 h. Data are presented as mean ± SD of three independent experiments (*p < 0.05,^**^p < 0.01 *vs.* H_2_O_2_; ^#^*p* < 0.05, ^##^*p* < 0.01 *vs.* Control).

As shown in **Figure 5A**, H_2_O_2_ stimulation markedly increased the levels of ROS in primary cortical neurons. However, treatment with CAT at doses of 12.5–50 μM led to marked inhibition of H_2_O_2_-induced expression of ROS (*p* < 0.01), indicating that CAT exerted good anti-oxidative effect.

**Figure 5B** showed that H_2_O_2_ significantly increased the expression of MDA, when compared with control group. Treatment with CAT at doses of 25–50 μM CAT significantly decreased the production of MDA (*p* < 0.05, *p* < 0.01). Besides, the level of GSH and SOD activity were markedly lowered after H_2_O_2_ treatment. However, CAT (25–50 μM) significantly increased GSH level and SOD activity (*p* < 0.01, **Figures 5C, D**).

### Effect of CAT on Apoptosis and MMP of Oxidative Stress-Injured Primary Cortical Neurons

**Figure 6:**
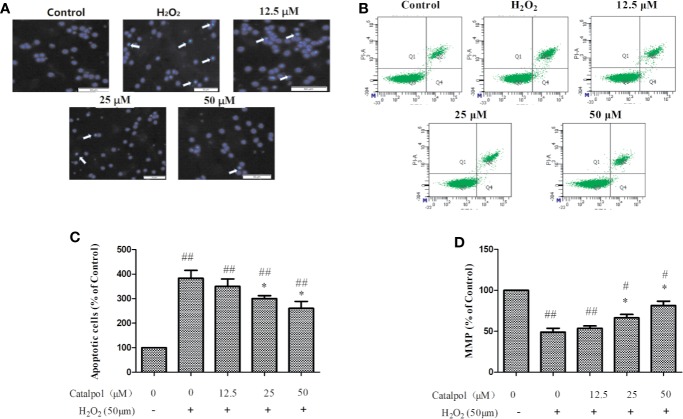
Apoptosis **(A–C)** and MMP **(D)** of H_2_O_2_-stimulated primary cortical neurons after treatment with CAT (12.5, 25, and 50 μM). Apoptosis was determined using DAPI staining (×50, arrow markers represent the apoptotic cells) and flow cytometry assays. Data are expressed as mean ± SD of three independent experiments (^*^p < 0.05 *vs.* H_2_O_2_; ^#^*p* < 0.05, ^##^*p* < 0.01 *vs.* control).

As shown in **Figure 6A**, apoptotic cells with aggregation of chromatin and nuclei were observed under a fluorescence microscope following DAPI staining in the H_2_O_2_ stimulation group. However, after treatment with CAT, the number of apoptotic cells decreased in a dose-dependent manner. The apoptotic cells were quantified using flow cytometry assays. **Figures 6B, C** show that the percentage of apoptotic cells (early and late apoptotic cells) were significantly higher in cells stimulated with H_2_O_2_, relative to the control group. However, CAT at doses of 25–50 μM, significantly reversed H_2_O_2_-induced apoptosis in primary cortical neurons (*p* < 0.05).

Quantitative measurement of MMP was done to determine cell death in primary cortical neurons stimulated with H_2_O_2_. As shown in **Figure 6D**, H_2_O_2_ significantly induced loss of MMP. However, treatment with CAT (25–50 μM) led to significant reversal of the H_2_O_2_-induced MMP reduction in primary cortical neurons (*p* < 0.05). Thus, the apoptosis of primary cortical neurons might have been caused by the loss of MMP function.

### Effect of CAT on Levels of Nrf2/HO-1 Pathway-Related Proteins in Primary Cortical Neurons

**Figure 7:**
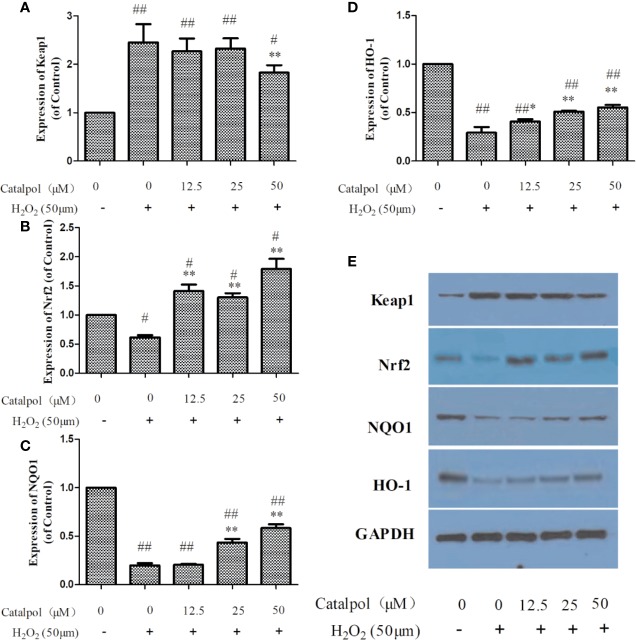
Effect of CAT (12.5, 25, and 50 μM) on the expressions of Keap1, Nrf2, NQO1 and HO-1 in oxidative stress-injured primary cortical neurons. The columns show quantification of Keap1 **(A)**, Nrf2 **(B)**, NQO1 **(C)**, and HO-1 **(D)**. Representative western blot image **(E)** showing the relative expressions of Keap1, Nrf2, NQO1, and HO-1 in the groups. Data are expressed as mean ± SD of three independent experiments (^*^p < 0.05, ^**^p < 0.01 *vs.* H_2_O_2_; ^#^*p* < 0.05, ^##^*p* < 0.01 *vs.* Control).

**Figure 7** displayed that H_2_O_2_ markedly decreased the expressions of Nrf2, NQO1, and HO-1, but increased the expression of Keap1 (*p* < 0.01). However, treatment with CAT significantly enhanced the protein levels of Nrf2, NQO1, and HO-1 (*p* < 0.05, *p* < 0.01), and lowered the protein level of Keap1 (*p* < 0.01).

### Effect of CAT on Levels of Apoptosis-Related Proteins in Primary Cortical Neurons

**Figure 8:**
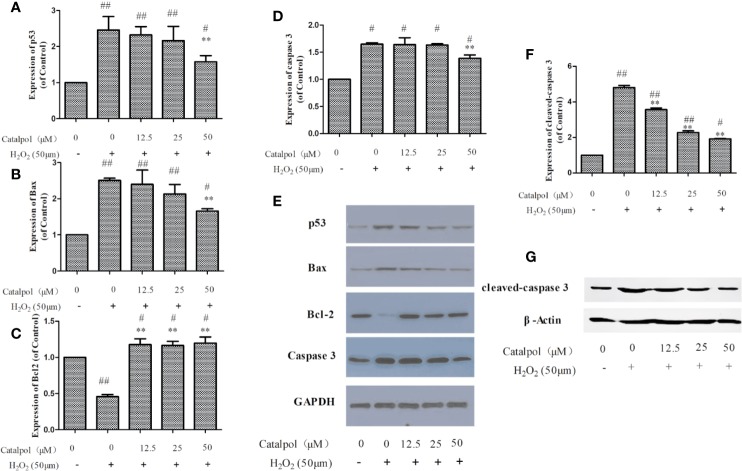
Effect of CAT (12.5, 25, and 50 μM) on the expressions of p53, Bax, Bcl2, caspase 3, and cleaved-caspase 3 in oxidative stress-injured primary cortical neurons. The columns show quantifications of p53 **(A)**, Bax **(B)**, Bcl2 **(C)**, caspase 3 **(D)**, and cleaved-caspase 3 **(F)**. Representative western blot images **(E, G)** show the expressions of p53, Bax, Bcl2, caspase 3, and cleaved-caspase 3 in the groups. Data are expressed as mean ± SD of three independent experiments (^**^p < 0.01 *vs.* H_2_O_2_; ^#^*p* < 0.05, ^##^*p* < 0.01 *vs.* control).

In order to further study the potential mechanism involved in the protection of the primary cortical neurons by CAT, the expressions of apoptosis-related proteins were measured with western blot. Results on **Figure 8** show that H_2_O_2_ markedly increased the expressions of p53, Bax, and caspase 3, but decreased the expression of Bcl-2 (*p* < 0.01). However, treatment with CAT significantly decreased the protein levels of p53, Bax, and caspase 3 (*p* < 0.05, *p* < 0.01), while the protein level of Bcl-2 was increased (*p* < 0.01). Since caspase 3 cleavage is a better indicator of apoptosis than an increase in total caspase 3, the expression of cleaved-caspase 3 was also assayed. Results showed that H_2_O_2_ significantly increased the expression of cleaved-caspase 3. However, CAT treatment (12.5, 25, and 50 μM) led to significant downregulations of the expression of cleaved caspase 3 (*p* < 0.01).

### Effect of CAT on Levels of NF-κB-Related Proteins in BV2 Microglia

**Figure 9:**
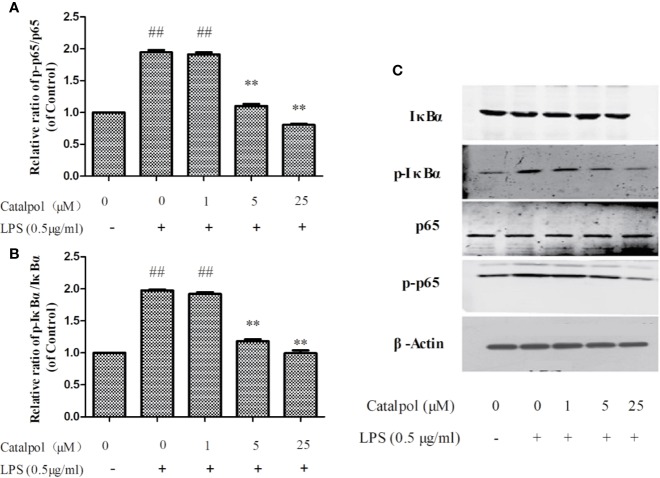
Effect of CAT (1, 5, and 25 μM) on the protein expression of NF-κB in BV2 cells. The columns show levels of p-p65/p65 **(A)** and p-IκBα/IκBα **(B)**. Representative western blot image **(C)** showing the expressions of IκBα, p-IκBα, p65, and p-p65 in all groups. Data are expressed as mean ± SD of three independent experiments (^**^p < 0.01 vs. LPS; ^##^*p* < 0.01 *vs.* control).

The potential mechanism involved in the protection BV2 microglia by CAT was investigated by assaying the expressions of *NF-κB*-related proteins using western blot. The results (**Figure 9**) showed that LPS markedly upregulated the value of p-IκBα/IκBα and p-p65/p65 (*p* < 0.01). However, treatment with CAT (5 and 25 μM) significantly downregulated the protein expressions of p-IκBα/IκBα and p-p65/p65 (*p* < 0.01).

## Discussion

Majority of nerve injuries are caused by complex of biochemical phenomena, such as protein aggregation, reactions of free radicals, glutamate excitotoxicity, inflammation, and oxidative stress. A damage such as nerve cell apoptosis or death is irreversible. It is thought that neuroprotective effects involve mechanisms such as inhibition of inflammation, excitotoxicity, oxidation, neuronal apoptosis, mitochondrial dysfunction, and calcium influx into cells (Tuttolomondo et al., 2015). In addition, neuro-inflammation and neuro-oxidation contribute to several neurodegenerative disorders, such as Parkinson's disease, Attention-deficit hyperactivity disorder, Huntington's disease, and Alzheimer's disease (McGeer and McGeer, 2004; Lopresti, 2015; Leffa et al., 2018; Li et al., 2020). Thus, it is necessary to study drugs that exert anti-oxidative and anti-inflammatory effects.

The complexity of structures in the brain make *in vivo* studies challenging. Cell lines derived from central nervous system precursors have limitations because the neurons derived from these lines fail to reflect the characteristics of central neurons, including the ability to form well-defined axons, dendrites, and synapses. In contrast, the primary cortical neurons reflect these characteristics. They have the advantages of single influencing factors, few interfering factors (such as blood circulation, body fluids, endocrine system, and blood-brain barrier), high repeatability, and ease of analysis of results. Therefore, primary cortical neurons were used as a model for *in vitro* studies (Reis, 2005). In this study, primary cortical neurons were stimulated with H2O2 to establish a cell model of oxidative stress damage. In the process of extracting primary cortical neurons, they become mixed up with many other cells such as astrocytes and vascular endothelial cells. Thus, differential adherence method and B27TM additive without OA were used to purify the cells (Xie et al., 2000). Neuronal cells were identified with double immunofluorescence staining. The results showed that the cell function was normal at the ninth day of culture and could be used for experiments.

SOD and GSH are predominant antioxidants involved in the neutralization of oxygen free radicals (Bose and Agrawal, 2006; Zhao et al., 2013). ROS are active oxygen radicals which directly reflect oxidative stress in cells. MDA reflects the severity of free radical attack, being the end product of lipid peroxidation in cell membranes. Thus, SOD, GSH, ROS, and MDA were used to assess the oxidative stress levels in primary cortical neurons. In this study, CAT increased the production of SOD and GSH, and decreased the production of MDA in H_2_O_2_-stimulated primary cortical neurons. Similar effects have been reported by other researches (Shu et al., 2016). For example, CAT increased the level of SOD and GSH, and decreased the level of MDA in the spleen and liver of aged mice (Zhang et al., 2008). In addition, results obtained in the present study showed that CAT at a concentration of 12.5 μM significantly reduced ROS levels but not MDA levels. There are two ways through which drugs exert antioxidant effects: direct clearance of free radicals, and production of antioxidant bio-molecules, for example GSH and SOD (Paulina et al., 2013). Thus, it is likely that CAT at low concentration directly acted on ROS, but failed to recover the endogenous antioxidant defense system (GSH and SOD), resulting in its failure to significantly reduce the production of MDA. There is a dynamic balance between ROS production and antioxidant defense system in the body. When this balance is impaired, oxidative stress response results. When stimulated with H_2_O_2_, cells tend to produce excessive ROS, which then cause apoptosis. It is generally believed that there are two mechanisms through which apoptosis may be induced: receptor-mediated pathway (exogenous pathway) and mitochondrial-dependent pathway (endogenous pathway). Mitochondrial-dependent apoptosis begins in the mitochondria and is regulated by endogenous ROS. Excessive ROS may act on different targets in this pathway. For example, ROS damage biomacromolecules in the mitochondrial membrane, leading to decreased MMP and opening of permeability transition pore, thereby deforming the mitochondrion. These events lead to further generation of ROS (a vicious cycle), and ultimately lead to apoptosis (Hanikoglu et al., 2019). The high level of ROS production leads to serious oxidative damage to DNA, activation of p53 and accumulation p53 in the nucleus (Harris and Levine, 2005). It has been reported that P53 induces apoptosis by regulating the expressions of apoptosis-related proteins such as Bcl-2 and Bax (Nkpaa et al., 2019). In normal cells, caspase-3 exists in an inactive state. When the cell is stimulated, caspase-3 is activated by enzymatic cleavage during apoptosis. Cleaved-caspase 3 is a better indicator of apoptosis than an increase in total caspase 3. Thus, the expressions of cleaved-caspase 3 and caspase 3 were determined in this study. Caspase-3 protein is a key downstream enzyme of the apoptotic pathway. It is involved in apoptosis in a variety of pathways, and it is considered to be the core enzyme that induces apoptosis. It was found that CAT inhibited H_2_O_2_-induced apoptosis of primary cortical neurons by regulating apoptosis-related proteins such as Bcl-2 and Bax. With H_2_O_2_ stimulation, the protein expressions of p53, Bax, caspase-3, and cleaved-caspase 3 were increased, while the expression of anti-apoptotic protein Bcl-2 was decreased. However, these protein expression profiles were reversed with CAT treatment.

It is known that Nrf2 is a transcription factor which participates in redox homeostasis by regulating the expressions of regulating anti-oxidative genes. It is present in most cell types of the brain, including neuronal cells, and it is a key member of the Keap1/Nrf2 signaling pathway. Indeed, it is likely that Nrf2 signaling is involved in the protection of neurons (Cuadrado et al., 2019). Under resting conditions, Nrf2 binds to cytoplasmic protein Keap1. When oxidative stress occurs, Nrf2 is phosphorylated, separated from Keap1, and transferred from the cytoplasm to the nucleus. Then, Nrf2 binds to the antioxidant response element (ARE) and up-regulates the expressions of NQO1, HO-1 and other factors, thereby exerting its anti-oxidative effect (Jin et al., 2015). In this study, the expression of Keap1 protein in the model group was significantly increased, while the expression of Nrf2 protein was significantly decreased. However, CAT decreased the expression of Keap1 and increased the expression of Nrf2, indicating that regulated the activation of Nrf2. It is also known that activated Nrf2 dissociates from Keap1, and is transferred from the cytoplasm to the nucleus and combines with the ARE to further induce the expression of HO-1, NQO1, and other proteins, thereby exerting anti-oxidative stress effects. Therefore, the Keap1-Nrf2-ARE signaling pathway, or part thereof, is involved in the antioxidant mechanism of CAT.

Microglia have common features with macrophages, and they have been implicated as predominant cells involved in regulation of inflammation-mediated neuronal damage. LPS is a thick layer of the outer membrane of gram-negative bacteria cell-wall. It plays an important role in chronic inflammation of the brain (Cuschieri and Maier, 2007). Microglia produces inflammatory mediators such as NO, as well as pro-inflammatory cytokines such as TNF-α and IL-6. These neurotoxic products induce neuronal cell damage and the production of nervous system disease (Bachiller et al., 2018; Hansen et al., 2018). In addition, pro-inflammatory cytokines trigger the production of ROS (Higashimoto et al., 2006). In the present study, pretreatment with CAT inhibited LPS-induced productions of NO, TNFα, and IL-6 in BV2 microglia through the NF-κB pathway. The NF-κBp65 is present in the cytoplasm of resting cells, and it combines with inhibitor IκBα. However, IκB protein regulated by IκB kinase complex in the cytoplasm is rapidly phosphorylated and degraded when NF-κB is activated by LPS, resulting in nuclear translocation of NF-κB dimers (Yun et al., 2008). The nuclear translocation of NF-κB dimers is involved in the expressions of genes linked to inflammation and apoptosis. The NF-κB is an important transcription factor which is expressed in brain cells, including neurons, microglia, and astrocytes, and it participates in several brain functions (Oneill and Kaltschmidt, 1997). It has been reported that NO regulates NF-κB transcription (Meffert and Baltimore, 2005). Moreover, it has been shown that CAT inhibited LPS and IFN-γ-induced inflammatory responses in astrocyte primary cultures (Bi et al., 2012). Thus, CAT exhibits neuroprotective effect by inhibiting neuro-inflammation.

**Figure 10:**
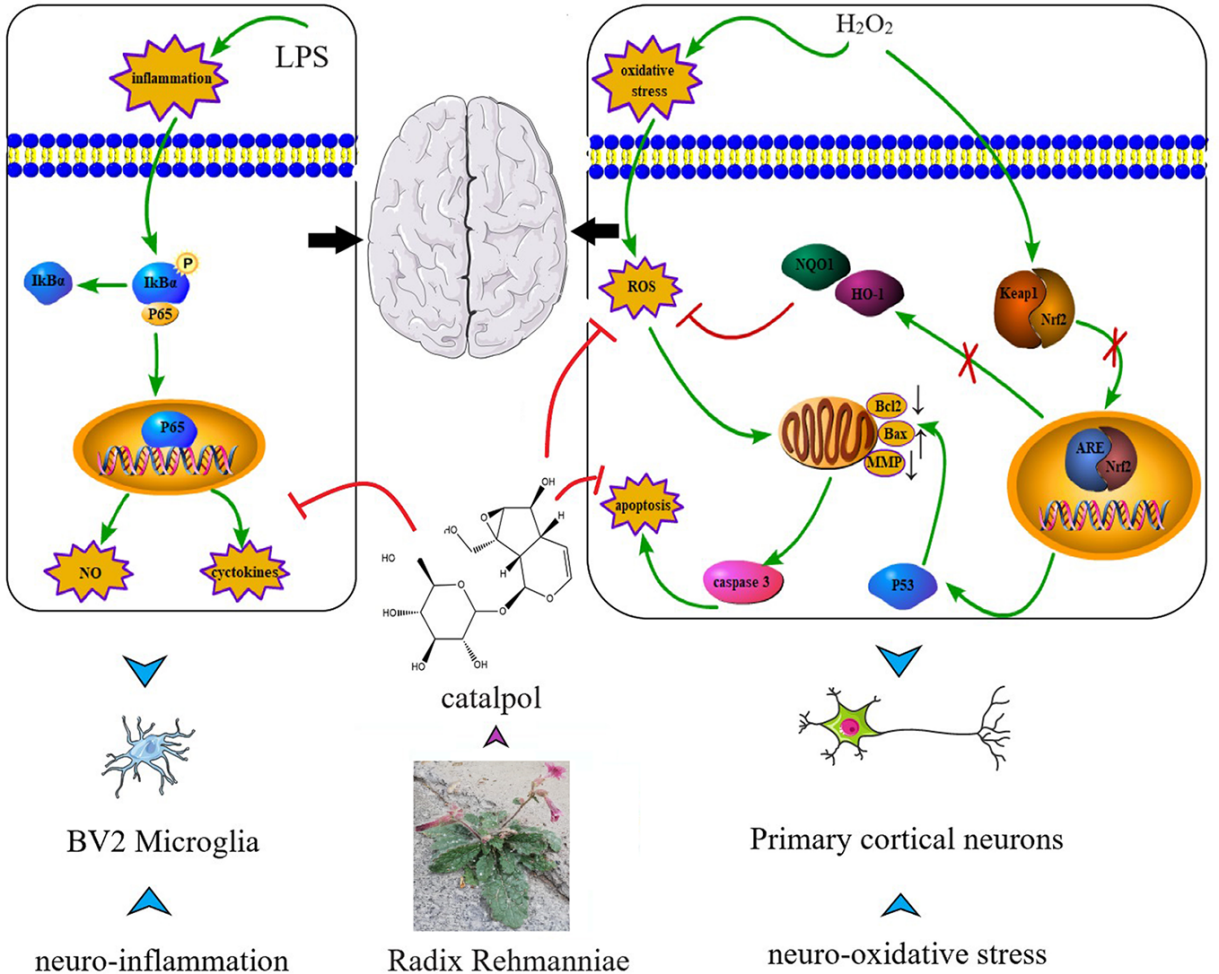
Schematic diagram of the targets of CAT.

Catalpol (CAT) has been studied extensively for its multiple pharmacological activities both *in vitro* and *in vivo*, including anti-diabetic, cardiovascular protective, neuroprotective, anticancer, hepatoprotective, anti-inflammatory, and anti-oxidant effects (Bai et al., 2019; Bhattamisra et al., 2019). Numerous studies have suggested the anti-inflammatory and anti-oxidative effects of CAT, such as in PC12 cells (Jiang et al., 2004), human umbilical vein endothelial cells (Hu et al., 2010), cardiac myocytes (Hu et al., 2016), and animal models of acute ischemic stroke (Zhu et al., 2015; Zheng et al., 2017). Although the pharmacological effects of CAT in the treatment of some diseases have been gradually discovered, its role and underlying mechanism in the treatment of neurological diseases are still unclear. Interestingly, in this study, we observed that CAT (particularly in higher dose) markedly downregulated the levels of NO, IL-6, and TNF-a in lipopolysaccharide (LPS)-treated BV2 microglial cells. Moreover, CAT significantly decreased the levels of ROS and MDA, increased SOD activity and GSH level, reversed apoptosis, and restored MMP in primary cortical neurons stimulated with H_2_O_2_. More importantly, NF-κB and p53-mediated Bcl-2/Bax/casaspe-3 apoptotic pathways were inhibited by CAT. Keap1/Nrf2 pathway was activated by CAT. These findings conclude that CAT has neuroprotective effect by attenuating microglial-mediated neuroinflammatory response and cortical neuronal oxidative damage (**Figure 10**). Therefore, CAT is a potential drug in the treatment of neurological diseases through activating the multi-signal pathways.

## Conclusion

The present study has demonstrated the neuroprotective effects of CAT (particularly in higher dose) in attenuating LPS-induced neuroinflammation *via* inhibition of the NF-κB signaling pathway in BV2 microglia, and in H_2_O_2_-induced oxidative stress through activation of the Nrf2/HO-1 signaling pathway and inactivation p53-mediated Bcl-2/Bax/casaspe-3 apoptosis pathway in primary cortical neurons. Due to the anti−inflammatory, anti-oxidative, and anti-apoptotic effects of CAT in nerve cells, CAT may be a potential agent for the treatment of neurological disease.

## Data Availability Statement

All datasets generated for this study are included in the article/supplementary material.

## Ethics Statement

The Animal Care and Use Committee of the Beijing Shijitan Hospital approved all animal protocols.

## Author Contributions

JN, DY, and CY designed the research. ZS, LY, and YD performed the experiments. JN, DY, and CY conducted the data analysis. All authors have reviewed and approved the final version of the manuscript.

## Funding

This work was supported by the Ministry of National Science and Technique (2014ZX09304306) and "Top youth team" of excellent talents in Beijing (2018000021223TD09).

## Conflict of Interest

The authors declare that the research was conducted in the absence of any commercial or financial relationships that could be construed as a potential conflict of interest.
